# Editorial: Functional Aspects of Mesoscopic Brain Oscillations: Insights From *in vivo* and *in vitro* Studies

**DOI:** 10.3389/fncir.2022.960157

**Published:** 2022-07-04

**Authors:** Gürsel Çalışkan, Sanja Mikulovic, Gabrielle Girardeau

**Affiliations:** ^1^Department of Genetics and Molecular Neurobiology, Institute of Biology, Otto-von-Guericke-University Magdeburg, Magdeburg, Germany; ^2^Center for Behavioral Brain Sciences, Magdeburg, Germany; ^3^Research Group Cognition and Emotion, Leibniz Institute for Neurobiology, Magdeburg, Germany; ^4^Research Group Sleep and Emotional Memory, Institut du Fer a Moulin, UMR-S 1270 INSERM and Sorbonne Université, Paris, France

**Keywords:** neural oscillations, cognition, innate and emotional behavior, memory, animal models

Oscillatory activity is considered a major way for the brain to convey information within and across structures. These patterns range from slow (<1 Hz) to fast oscillations (>200 Hz) in a brain-state- and circuit-dependent manner (Buzsáki and Draguhn, 2004). From a functional perspective, a major challenge is to elucidate how a given frequency in a given brain circuit is associated with a specific behavior and try to understand how the identified oscillations sustain the cognitive processes associated with this state or behavior (Lee and Dan, 2012). In recent years, there has been a major leap in deciphering the mechanisms of distinct mesoscopic brain rhythms and their utilization for treatment of diseased brain states associated with aberrant emotional and cognitive behavior. In this Research Topic, we present a collection of 10 articles (3 Original Research, 1 Methods, 4 Reviews, 1 Mini Review, and 1 Perspective) that highlight recent advances on mechanisms underlying mesoscopic brain rhythms and their function in health and disease.

In their perspective article, Nokia and Pentonnen discuss the foundational and more recent work that led to the classical trio of brain oscillations for episodic memory consolidation: ripples, spindles and slow oscillation (SO). They underline the importance of two additional patterns that have recently emerged as strong modulators memory consolidation mediated by ripple-spindle-SO coordination: the historically understudied hippocampal dentate spike and breathing. The latter highlights the recent and growing body of evidence linking bodily parameters such as breathing and heart rate to cognitive processes.

In this line, Folschweiller and Sauer elaborate on the generation mechanisms and function of respiration-driven brain activity with a particular attention to its role in emotional cognition (e.g., fear, reward, despair) across species. They further discuss how breathing can impact cognition *via* modulation of specific oscillatory patterns [e.g., gamma (γ) oscillations and ripples] and underlying neuronal ensembles.

Aguilera et al. mini review addresses hippocampal theta (θ)-γ dynamics and suggests that the current model, focusing on discrete subtypes of γ activity, does not capture its full complexity. The authors refer to the recent studies showing that many θ cycles contain multiple γ bouts of various frequencies and propose application of novel methods, such as machine learning approaches, in order to extract the meaningful behavior-relevant information, without relying on pre-defined γ subtypes.

In their review article, Ibarra-Lecue et al. take a very broad perspective on brain rhythms and retrace the history of the study of brain oscillations up to the most recent findings. They illustrate a gradual shift from correlational studies to causal ones with the advent of new technologies such as optogenetics, while highlighting both the promises and limitations of these approaches to circuit and function dissection.

Földi et al. focuses on the neural mechanisms giving rise to “oscillopathies,” with a particular focus on epileptic seizures. They discuss the importance of designing reliable seizure-predicting algorithms and devices capable of predicting and preventing seizures from happening. In particular, they put forward the idea of applying closed-loop techniques for “oscillotherapeutics” and refer, among others, to their own recent work in which closed-loop stimulation of the medial septum was used to successfully terminate epileptic seizures in rats.

The article by Speers and Bilkey reviews the vast literature on the abnormalities observed in θ, γ, and ripple oscillations in animal models of schizophrenia and discusses these findings with respect to studies in human subjects. They particularly focus on the disruption of hippocampal θ-phase precession, an important phenomenon for the sequential activation and reactivation of place cells, as a potential mechanism underlying cognitive deficits in schizophrenia.

Of note, specific oscillatory frequency ranges are used for the automatic classification of brain states or behaviors, together with the movement information (muscular activity and/or velocity). However, some behaviors are associated with oscillatory bands that overlap with each other. Notably, freezing and resting epochs including REM (Rapid Eye Movement) sleep, Non-REM sleep and quiet wakefulness are all characterized by immobility and overlapping oscillations. In their method paper, Pompili and Todorova establish that classical methods fail to discriminate between freezing and rest, and propose a novel tool using cortical spindles, that can be recorded with a simple EEG surface electrode, to reliable disambiguate freezing from non-REM sleep. This can be especially useful for studies using fear conditioning and extinction to study memory formation.

Neuromodulation is classically associated with long time scales. In their original article, Jelitai et al. however describe an inhibitory subpopulation in the serotoninergic median raphe region whose activity is coupled to hippocampal theta oscillations and ripples. These neurons synchronize ascending serotonergic and glutamatergic modulation with hippocampal activity on a subsecond timescale. The authors suggest that short and long timescale modulation are controlled by different mechanisms and open space for future studies investigating modulation with high temporospatial resolution.

Two original research articles use *in vitro* oscillation models in rodent hippocampal slice preparations. Such *in vitro* models provide robust tools for interrogating underlying cellular mechanisms and pharmacology of network oscillations. Landeck et al. demonstrate that enriched environments reduce inhibition during spontaneous hippocampal sharp wave ripples (SWR), leading to enhanced but disorganized neuronal activity during SWR. These observations suggest that *in vitro* oscillations are sensitive to behavioral manipulations and can be used as proxies for metaplasticity in the hippocampal sub-circuits. On the other hand, Klemz et al. perform pharmacological experiments on cholinergic-induced gamma oscillations using blockers or activators of several channels important for modulation of intrinsic cellular excitability. They show specific changes in gamma oscillation power upon modulation of voltage- and calcium-activated ion channels, highlighting their potential use against cognitive impairment.

With this Research Topic, we have aimed to catalyze the collective effort on identification of functional aspects of brain oscillatory activities and promote the use of these rhythms as biomarkers. We believe that the development of “oscillotherapeutics” will help correcting circuit dysfunctions underlying with disturbed emotional and cognitive functions in various psychiatric diseases.

## Author Contributions

GÇ, SM, and GG prepared and discussed a list of guests-authors, invited them, revised their manuscripts, and handled their revisions. The Editorial was written by all authors. All authors contributed to the article and approved the submitted version.

## Conflict of Interest

The authors declare that the research was conducted in the absence of any commercial or financial relationships that could be construed as a potential conflict of interest.

## Publisher's Note

All claims expressed in this article are solely those of the authors and do not necessarily represent those of their affiliated organizations, or those of the publisher, the editors and the reviewers. Any product that may be evaluated in this article, or claim that may be made by its manufacturer, is not guaranteed or endorsed by the publisher.
